# Gender differences in health related quality of life of young heroin users

**DOI:** 10.1186/1477-7525-8-145

**Published:** 2010-12-01

**Authors:** Antònia Domingo-Salvany, M Teresa Brugal, Gregorio Barrio, Francisco González-Saiz, M José Bravo, Luís de la Fuente

**Affiliations:** 1 Drug Abuse Epidemiology Research Group. IMIM-Hospital del Mar. Dr. Aiguader, 88. E-08003 Barcelona, Spain; 2 CIBER de Epidemiología y Salud Pública (CIBERESP), Spain; 3 Public Health Agency (ASPB). Pl Lesseps 1. E-08023. Barcelona, Spain; 4 Escuela Nacional de Sanidad. Avenida Monforte de Lemos 5. 28029-Madrid, Spain; 5 Fundación Andaluza para la Atención e Incorporación Social (FADAIS). Avda. de Hytasa, edificio Toledo II. Plt., 3ª, Ofic. n° 1. E-41006 Sevilla, Spain; 6 Centro Nacional de Epidemiología. Instituto de Salud Carlos III. Sinesio Delgado 6. Madrid, Spain

## Abstract

**Background:**

Health Related Quality of Life (HRQL) of opiate users has been studied in treatment settings, where assistance for drug use was sought. In this study we ascertain factors related to HRQL of young opiate users recruited outside treatment facilities, considering both genders separately.

**Methods:**

Current opiate users (18-30 y) were recruited in outdoor settings in three Spanish cities (Barcelona, Madrid, Sevilla). Standardised laptop interviews included socio-demographic data, drug use patterns, health related issues, the Severity of Dependence Scale (SDS) and the Nottingham Health Profile (NHP).

**Results:**

A total of 991 subjects (73% males), mean age = 25.7 years were interviewed. The mean global NHP score differed by gender (women: 41.2 (sd:23.8); men:34.1(sd:23.6);p < 0.05). Multivariate analysis was implemented separately by gender, variables independently related with global NHP score, both for males and females, were heroin and cocaine SDS scores. For women, only other drug related variables (alcohol intake and length of cocaine use) were independently associated with their HRQL. HIV+ males who suffered an opiate overdose or had psychiatric care in the last 12 months perceived their health as poorer, while those who had ever been in methadone treatment in the last 12 months perceived it as better. The model with both genders showed all factors for males plus quantity of alcohol and an interaction between gender and HIV status.

**Conclusions:**

Heroin users were found to be at a considerable risk of impaired HRQL, even in these young ages. A score approaching severity of dependence was the factor with the strongest relation with it.

## Background

Although some changes seem to be taking place in the incidence trends of specific illegal drugs, heroin use is still an important health concern in Europe. In most countries heroin remains the principal drug involved in treatment episodes[1] and heroin users are at a greater risk of dying from different causes, particularly overdoses but also infectious diseases related to injection[2-4].

Health Related Quality of Life (HRQL) has progressively been applied in the evaluation of health status of patients, including substance users[5,6]. Poor HRQL has been reported among heroin users starting treatment, being comparable to other chronic disease patients[7-9]. As a patient centred outcome variable, HRQL has also been used to assess treatment effectiveness and in randomised trials providing evidence of HRQL improvement with opioid substitution therapies [10-13]. Variables that have been related to poorer HRQL in opiate users vary in different studies. The more consistent finding is poorer HRQL associated with poly-drug use, HRQL has also been related to socio-demographic variables such as age, educational level or employment status, and the presence of chronic medical conditions, including HIV infection[8,14]. Although gender has been associated with differences in HRQL in many different population studies, being poorer in women[15,16], no clear differences have been reported in studies on opiate users [8,17,18]. The influence of psychiatric diagnoses other than substance use disorders on HRQL has been explored, results being inconsistent though mainly showing impaired HRQL in subjects with dual diagnosis[18-20]. It is difficult to compare the various studies as they have explored different variables and used different HRQL measures. The generic HRQL measures most frequently used have been the SF-36 and the Nottingham Health Profile (NHP). The German adaptation of the Lancashire Quality of Life Profile, a questionnaire designed specifically for the mental health field, has also been used in studies with drug users [13,21]. Few HRQL instruments specific to the drug dependence field are available[22].

Episodes of drug overdose are frequent among heroin injectors[23,24] and it has been suggested that poor health may be an important overdose risk factor[25,26], yet we don't know of any previous study exploring the possible relation between perceived HRQL and overdose experiences which could be of interest for specific prevention. It is possible that HRQL is being affected in early phases of opiate use, however as far as we know there is little information on HRQL in young opiate users, early in their drug career. Most studies have been done after entry to treatment.

The objective of the present study was to ascertain what factors were related with HRQL among young opiate users, including previous drug treatment and overdose episodes, taking gender into account.

## Methods

The ITINERE project cohort of current regular users of heroin aged between 18 and 30 years was assembled in outdoor settings of three Spanish cities (Barcelona, Madrid, Sevilla). Details of the methodology have been described previously [24,27]. To be included, subjects had to be residents in the above mentioned cities, to have used heroin within the 90 days prior to the interview, and at least 12 days over the 12 months prior to the interview; they also had to be willing to participate in and facilitate the follow-up. Exclusion criteria were language barriers and difficulties in follow-up. For recruitment, targeted sampling and nomination techniques, with different starting points mainly in outdoor locations, was used[28]. After a brief selection questionnaire, to assess fulfilment of inclusion criteria, candidates were informed about the objectives and procedures of the study, including incentives for participation (18 Euro per interview completed) and signed an informed consent. Field work was done between April 2001 and December 2003. The inception cohort baseline questionnaire was administered through a laptop assisted interview in socio-sanitary premises and included, among other variables, socio-demographic data, drug use patterns, health problems data, severity of heroin and cocaine dependence measured through the Spanish version of the Severity of Dependence Scale (SDS)[29,30], and a generic health related quality of life questionnaire, the Nottingham Health Profile (NHP)[31]. Interviewers were trained social science professionals (i.e.: anthropologists, sociologists,...).

A non-fatal opiate overdose was defined as an episode occurring after heroin or opiate use characterized by extreme difficulty in breathing, loss of consciousness and problems waking up or recovering consciousness, and possibly bluish skin or lips. Other variables studied were having been confined to bed due to discomfort, disease or injury, on any day during the last 12 months and to have been in hospital as an inpatient during the same period. The use of two or more illegal substances during the last 12 months with a frequency of once weekly or higher was considered a proxy of poly-drug use. Alcohol consumption was measured as intake in grams/day and categorized in 4 risk categories (no use, moderate, at-risk and heavy) with different cut-points by gender (male 40 and 60 g/day, female 20 and 40 g/day). Serological tests (HIV, HBV, HCV) were done through a dried blood spot test. The ITINERE project has been approved by the ethical committee of the Instituto de Salud Carlos III.

The SDS is a short, easily administered scale which can be used to measure the degree of dependence experienced by users of different types of drugs. The SDS contains five items, all of which are explicitly concerned with impaired control over drug taking and with worries and anxieties about drug use. It satisfies a number of criteria indicating its suitability as a measure of dependence[29]. It was applied to assess dependence severity (range 0, none - 15, most) for heroin (SDS-H) and for cocaine (SDS-C).

The Nottingham Health Profile (NHP) is a multidimensional health status questionnaire that has been previously used in drug users[10,11] and found to be easy to administer in this population. It contains 38 items divided into 6 dimensions of health (energy, pain, sleep, social isolation, emotional reactions, physical mobility) each one scored from 0, best to 100, worst health state. A global NHP score was calculated taking the mean of the six dimension scores. To compare the study results to the general population we used NHP Spanish norms for ages 41 to 49. There is no normative data available for younger ages but as from HRQL studies we know that generic HRQL scores are better for younger age groups[31], if appropriate age specific reference values were to have been used, differences potentially found would have been even larger.

Differences by gender were tested using chi-square test or t-test. To compare possible differences in NHP scores, non parametric tests (Mann-Witney U or Kruskal-Wallis test-with correction for ties, if necessary) were used. As large samples were analysed, for multivariate analysis the NHP global score was considered as normally distributed[32] and a multiple linear regression applied. All variables significant or marginally significant (p < 0.10) in bivariate analysis were included in three models, one for the total and one per gender, and the selection of final variables was done with a backward procedure. All analyses were done with SPSS 12.0.

## Results

A total of 991 young heroin users were recruited, 722 were male (73%) and 269 female. Men and women differed in all socio-demographic variables explored, but also in some general health (confined to bed at least one day in the last 12 months, HIV positive: more frequent in women) and drug use variables (a higher proportion of heavy alcohol use, and a shorter length of heroin and cocaine use among women)(table 1). No gender differences were observed in the proportion of those who had a previous overdose experience or had experienced an opiate overdose in the last 12 months. However, the proportion of those who had recently (12 months) experienced a non-fatal overdose (n = 80) was higher in Barcelona, among those more educated, squatters or homeless, unemployed, those who had been in hospital in the last 12 months, were anti-HCV positives, had injected in the last 12 months, or had not been in methadone treatment at any time in the last 12 months.

**Table 1:** Socio-demographic variables and drug use patterns, in the overall sample and by gender.

	Women 269 (27%) n (%)	Men 722 (73%) n (%)	Total 991 n (%)	p
**Age **(mean; [s.d.])	25.00 [3.6]	25.9 [3.2]	25.7 [3.3]	< 0.0001
**Educational level**				0.008
Primary or <	104 (38.8)	348 (48.2)	450 (45.7)	
> = Secondary	164 (61.2)	370 (51.8)	530 (54.3)	
**Living arrangements **^†^				0.007
Flats	187 (69.5)	507 (70.2)	694 (70.0)	
Squats	56 (20.8)	103 (14.3)	159 (16.0)	
Homeless or institution	26 (9.7)	112 (15.5)	138 (13.9)	
**Work**				
Did not work ^†^ (with/without contract)	203 (75.5)	475 (65.8)	678 (68.4)	0.004
**Ever in prison**	71 (26.4)	347 (48.1)	418 (42.2)	< 0.0001
**Ever confined to bed ^†^**	143 (54.0)	272 (38.0)	415 (42.3)	< 0.0001
**Inpatient in a hospital **^†^	65 (24.5)	142 (19.8)	207 (21.1)	0.107
**Infections **(n = 971)				
Ab* HIV +	61 (22.9)	116 (16.5)	177 (18.2)	0.020
Ab HCV +	132 (49.6)	375 (53.3)	507 (52.3)	0.311
Ab HBV core +	41 (15.4)	124 (17.6)	165 (17.0)	0.421
**Alcohol use severity‡**				0.004
No alcohol use	64 (23.9)	113 (15.8)	177 (18.0)	
Moderate	95 (35.4)	317 (44.3)	412 (41.9)	
At risk	41 (15.3)	133 (18.6)	174 (17.7)	
Heavy use	68 (25.4)	152 (21.3)	220 (22.4)	
**N of years drug use **(mean; [s.d.])				
Cocaine	8.2 [4.0]	9.6 [3.9]	9.2 [4.0]	< 0.0001
Heroin	7.4 [4.6]	8.9 [4.3]	8.5 [4.5]	< 0.0001
**Poly-drug use **^†^	255 (94.8)	686 (95.0)	941 (95.0)	0.889
**Ever injecting**	164 (61.0)	473 (65.5)	637 (64.3)	0.184
**Age first heroin use **(mean; [s.d.])	17.6 [3.4]	17.0 [3.1]	17.1 [3.2]	0.001
**Age first injecting **(mean; [s.d.])	19.6 [3.7]	19.3 [3.9]	19.4 [3.8]	0.392
**Intravenous use **^†^	135 (50.6)	381 (52.9)	516 (52.3)	0.511
**Drug use treatment**				0.291
Never	84 (31.7)	213 (29.7)	297 (30.2)	
Before last year	40 (15.1)	141 (19.7)	181 (18.4)	
Methadone last year	108 (40.8)	262 (36.5)	370 (37.7)	
Other last year	33 (12.5)	101 (14.1)	134 (13.6)	
**Psychiatric treatment **^†^	25 (9.3)	51 (7.1)	76 (7.7)	0.241
**Opiate Overdoses**				
Ever in lifetime	71 (26.5)	173 (24.0)	244 (24.6)	0.412
Last 12 months	22 (8.2)	58 (8.0)	80 (8.1)	0.928
SDS * score (mean; [s.d.])				
Cocaine	5.3 [4.3]	4.8 [4.1]	4.9 [4.2]	0.089
Heroin	8.2 [3.3]	8.0 [3.4]	8.1 [3.4]	0.497

* Ab: antibodies; SDS: Severity Dependence Scale.

^† ^Refers to last 12 months.

^‡ ^different cut-points used for both genders: men: 40-60 g/day; women: 20-40 g/day

**Figure 1:**
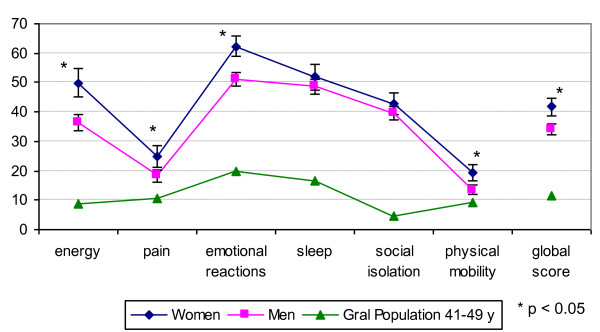
**Dimensions of the Nottingham Health Profile (NHP) and global NHP score by gender, compared to the general population profile**[31].

A valid NHP questionnaire was obtained for 963 subjects, 97% of the sample. The mean global NHP score was 36.0 (sd: 23.8). Women perceived their health as worse than men in all dimensions (global score: 41.2 (23.8) vs 34.1 (23.6)) (Figure 1), though not statistically significant for sleep and social isolation. In all dimensions NHP scores were higher for both genders than those of the general population (NHP global score in general adult population 41-49 years old: 11.0 (sd:13.6)). NHP global score was higher in older ages with a significant positive correlation in both genders. The NHP global score showed statistically significant differences in both genders according to current employment (better), living arrangements (better among squatters) and prison experience (worse). It was also worse with longer duration of heroin use and with higher scores for SDS-H and SDS-C. Among males it was poorer in lower educational levels, those who were ever confined to bed or visited a psychiatrist during the previous 12 months, were HIV positive, had core antibodies of hepatitis B, or had ever had an overdose. Among women it was poorer with increased length of cocaine use (table 2). NHP global score showed statistically significant differences for poly-drug use and hospital inpatient admission in the last 12 months (worse in affirmative categories), only when considering both genders simultaneously.

**Table 2:** Global NHP score (95% Confidence Interval) by gender in different socio-demographic, drug use and health related variables.

	Women	Men	Total
	N = 262	N = 701	N = 963
	**Mean (95% CI)**	**Mean (95% CI)**	**Mean (95% CI)**

**All**	41.2 (38.3-44.1)	34.1 (32.4-35.8)	36.0 (34.5-37.5)
**Age groups**	*		
18-24 y	35.9 (32.4-39.4)	31.4 (28.3-34.5)	32.9 (30.5-35.3)
25-28 y	45.1 (39.4-50.8)	35.3 (32.6-38.0)	37.5 (35.0-40.0)
29-30 y	45.4 (39.1-51.7)	35.1 (31.8-38.4)	37.8 (34.8-40.8)
**Educational level**		**	**
Primary or <	45.0 (40.0-50.0)	38.4 (35.8-41.0)	39.9 (37.6-42.2)
> = Secondary	38.8 (35.4-42.2)	29.9 (27.7-32.1)	32.7 (30.8-34.6)
**Living arrangements †**	*	*	*
Flats	40.6 (37.1-44.1)	35.1 (33.0-37.2)	36.6 (34.8-38.4)
Squats	37.9 (32.2-43.6)	27.0 (23.1-30.9)	30.9 (27.6-34.2)
Homeless or institution	52.0 (43.0-61.0)	35.8 (31.6-40.0)	38.9 (34.9-42.9)
**Work †**	*	*	**
Yes	33.6 (27.8-39.4)	30.9 (28.1-33.7)	31.5 (28.9-34.1)
Did not work	43.7 (40.5-46.9)	35.7 (33.5-37.9)	38.1 (36.3-39.9)
**Ever in prison**	*	*	*
No	38.9 (35.6-42.2)	31.5 (29.2-33.8)	34.1 (32.2-36.0)
Yes	47.4 (41.7-53.1)	36.8 (34.2-39.4)	38.6 (36.2-41.0)
**Ever confined to bed †**		*	**
No	39.8 (35.5-44.1)	31.6 (29.5-33.7)	33.4 (31.5-35.3)
Yes	42.1 (38.2-46.0)	37.9 (34.9-40.9)	39.3 (36.9-41.7)
**Inpatient in a hospital †**			*
No	40.1 (36.8-43.4)	33.3 (31.3-35.3)	35.1 (33.4-36.8)
Yes	43.8 (37.8-49.8)	37.0 (33.1-40.9)	39.2 (35.9-42.5)
**Infections**		*	*
Abª HIV -	40.4 (37.2-43.6)	33.0 (31.1-34.9)	34.9 (33.2-36.6)
Ab HIV +	43.4 (37.1-49.7)	39.9 (35.6-44.2)	41.1 (37.5-44.7)
			
Ab HCV -	39.2 (35.4-43.0)	32.7 (30.1-35.3)	34.6 (32.4-36.8)
Ab HCV +	42.9 (38.6-47.2)	35.4 (33.0-37.8)	37.4 (35.4-39.6)
		*	
Ab HBV core -	40.9 (37.8-44.0)	33.2 (31.3-35.1)	35.3 (33.7-36.9)
Ab HBV core +	41.9 (34.2-49.6)	38.7 (34.3-43.1)	39.5 (35.7-43.3)
			
**Alcohol use severity‡**	*		
No alcohol use	35.9 (30.0-41.8)	37.6 (32.9-42.3)	37.0 (33.3-40.7)
Moderate	40.5 (35.5-45.5)	31.8 (29.3-34.3)	33.7 (31.5-35.9)
At risk	39.6 (32.5-46.7)	35.9 (30.0-41.8)	37.3 (32.7-41.9)
Heavy use	47.5 (42.0-53.0)	34.8 (31.6-38.0)	37.8 (35.0-40.6)
**N of years drug use**	*	*	*
Cocaine			
0-4 y	35.7 (30.3-41.1)	30.0 (25.6-34.4)	32.1 (28.7-35.5)
5-10 y	39.1 (35.1-43.1)	32.7 (29.9-35.5)	34.7 (32.4-37.0)
> 10 y	48.2 (42.5-53.9)	36.4 (33.8-39.0)	38.8 (36.4-41.2)
Heroin		*	*
0-4 y	36.8 (32.2-41.4)	28.7 (25.3-32.1)	31.7 (28.9-34.5)
5-10 y	40.9 (36.5-45.3)	33.4 (30.6-36.2)	35.5 (33.1-37.9)
> 10 y	46.3 (40.3-52.3)	37.2 (34.4-40.0)	39.1 (36.5-41.7)
**Poly-drug use†**			*
No	30.0 (17.4-42.6)	29.2 (19.9-38.5)	29.4 (21.9-36.9)
Yes	41.8 (38.9-44.7)	34.3 (32.5-36.1)	36.3 (34.8-37.8)
**Injection**			
Never	39.7 (35.0-44.4)	33.3 (30.3-36.3)	35.2 (32.6-37.8)
Ever injecting	42.1 (38.4-45.8)	34.5 (32.4-36.6)	36.4 (34.5-38.3)
			
Intravenous use last 12 m	41.4 (37.4-45.4)	34.9 (32.6-37.2)	36.6 (34.6-38.6)
**Drug use treatment**			
Never	38.0 (32.85-43.15)	31.5 (28.1-34.9)	33.4 (30.6-36.2)
Before last year	43.7 (37.42-49.98)	34.4 (30.7-38.1)	36.4 (33.2-39.6)
Methadone last year	43.1 (38.38-47.82)	35.8 (32.8-38.8)	38.0 (35.4-40.6)
Other last year	38.5 (30.08-46.92)	34.2 (30.1-38.3)	35.3 (31.5-39.1)
**Psychiatric treatment †**		*	*
No	40.6 (37.58-43.62)	33.4 (31.6-35.2)	35.3 (33.7-36.9)
Yes	46.4 (36.68-56.12)	42.9 (36.0-49.8)	44.1 (38.5-49.7)
**Opiate overdoses**		*	*
Never	41.0 (37.58-44.42)	32.7 (30.7-34.7)	34.9 (33.1-36.7)
Ever	41.7 (36.34-47.06)	38.5 (35.1-41.9)	39.4 (36.5-42.3)
			
Last 12 months	43.2 (33.23-53.17)	37.9 (31.3-44.5)	39.4 (33.9-44.9)
**SDSª score**	**	**	**
Cocaine			
0 - 1	32.2 (27.58-36.82)	25.1 (22.3-27.9)	26.9 (24.5-29.3)
2 - 6	40.5 (35.6-45.4)	33.7 (30.9-36.5)	35.4 (33.0-37.8)
7 - 15	47.5 (42.78-52.22)	42.3 (39.2-45.4)	43.9 (41.3-46.5)
Heroin	**	**	**
0 - 6	32.3 (27.5-37.1)	23.9 (21.3-26.5)	26.1 (23.8-28.4)
7 - 9	36.6 (32.48-40.72)	34.8 (31.8-37.8)	35.3 (32.8-37.8)
10 - 15	52.4 (47.48-57.32)	42.1 (39.2-45.0)	44.9 (42.4-47.4)

ª CI: Confidence Interval; Ab: antibodies; SDS: Severity of Dependence scale

* p < 0.05; **p < 0.001

† refers to last 12 months

‡ different cut-points used for both genders: men: 40-60 g/day; women: 20-40 g/day

Having had an opiate overdose in the last 12 months, though it was not significant in bivariate analysis was included in the multivariate analysis instead of overdose ever, statistically significant in males but too remote from HRQL assessment. In males, the final multiple linear regression model, adjusted for age, showed that NHP global score was associated with socio-demographic variables (level of education, living arrangements, current employment), was impaired with some medical (ever confined to bed in the previous 12 months, HIV positive) and drug use related variables: higher scores on severity of heroin and cocaine dependence (SDS-H and SDS-C) and having experienced an opiate overdose in the last 12 months; and while it was worse in those men that had visited a psychiatrist in the previous 12 months, for those ever on methadone treatment in previous 12 months it was better (Table 3). Variables included in the regression explained 22.7% of the NHP global score variance. The severity of heroin dependence, as a continuous variable, showed the highest standardized beta coefficient (0.26). An increase of one point in the score of SDS-H was associated with an increase of 1.8 points in the NHP global score, while having an overdose during the previous 12 months increased it by 7 points. For females, only drug use related variables (daily alcohol intake, length of cocaine use and SDS-H and SDS-C) were independently related to global NHP score, explaining also 22.7% of the NHP global score variance. An increase of one point in SDS-H was associated with an increase of 2.1 points in the NHP global score (Table 3). When analysing the overall sample, all variables significant for males were included in the model plus daily alcohol intake, significant for females; however the regression involved an interaction term between gender and HIV status showing that women had worse NHP score which was not modified by their HIV status, whereas among men NHP score was impaired when HIV positive (Table 3).

**Table 3:** Multiple linear regressions, by gender, with Global NHP score as the dependent variable.

	Women			Men			Total		
	N = 262			N = 680			N = 929		
	**beta**	**Standardised beta**	**p value**	**beta**	**Standardised beta**	**p value**	**beta**	**Standardised beta**	**p value**

Constant	13.302		0.183	11.763		0.094	15.690		0.007
Men							-7.628	-0.144	0.000
Age	-0.218	-0.033	0.648	0.246	0.033	0.380	0.279	0.039	0.231
Work †				-5.095	-0.103	0.003	-5.056	-0.100	0.001
Educational level > = Secondary				-6.251	-0.133	0.000	-5.509	-0.116	0.000
Living arrangements † (ref.:flats)									
Squats				-6.255	-0.093	0.012	-5.025	-0.078	0.016
Homeless or institution				-1.608	-0.025	0.495	0.004	0.000	0.998
Ever confined to bed †				5.728	0.119	0.001	5.101	0.106	0.000
SDS* Heroin	2.144	0.301	0.000	1.813	0.259	0.000	1.899	0.268	0.000
SDS Cocaine	1.049	0.188	0.001	1.264	0.221	0.000	1.209	0.212	0.000
Length cocaine use	0.915	0.153	0.033						
Drug treatment (ref.: never)									
Before last year				-3.114	-0.053	0.223	-1.963	-0.032	0.374
Methadone last year				-5.622	-0.115	0.014	-4.880	-0.100	0.012
Other last year				-4.244	-0.063	0.125	-2.590	-0.037	0.275
Psychiatric treatment †				9.330	0.100	0.004	7.330	0.082	0.005
Overdoses †				7.113	0.082	0.019	5.413	0.061	0.038
HIV+				8.356	0.132	0.000	-0.788	-0.013	0.805
Alcohol consumption (g/day)	0.055	0.200	0.000				0.023	0.080	0.007
Men * HIV +							9.160	0.124	0.016
Adjusted R2	0.227			0.227			0.236		

* SDS: Severity of Dependence Scale

† last 12 months

## Discussion

HRQL was found to be impaired in young heroin users recruited outside the healthcare context, and severities of heroin and cocaine dependence were the variables that accounted for most of its explained variability in both genders. Women reported worse HRQL, but contrary to males having had an opiate overdose, contact with a psychiatrist or having ever been on methadone treatment during the preceding 12 months were not found to be associated with it.

A large sample was assembled that allowed to study a wide set of variables and to explore characteristics among women separately. It was planned to include young users to study the course of heroin use, trying to recruit users in early phases of their drug career and, in fact, they were younger than heroin users when requesting first treatment in Spain (mean age in 2002: 31.8 years)[33], however, the final sample included young heroin users already very much involved in heroin use. As elsewhere, it is difficult to ascertain the degree of representativeness of the population of young heroin users in the three cities where the study was conducted. Even though strategies to include users from different surroundings in the cities were implemented the final sample was somewhat biased towards heavy use. Another limitation of the present study could be related to the assumption of normality of the NHP global score. However, according to Lumley et al [32] the fact of being a large sample minimizes this problem. Furthermore, only 2.5% of participants presented a score of 0, suggestive of a floor effect, which can be considered as negligible. Also, when interpreting results it is necessary to remember that the cross-sectional nature of the study precludes making causal inferences in most of the variables.

The variables that explained most of the global NHP score variability were the same in both genders: the SDS-H and SDS-C accounted for 55.9% of the explained variance in women and for 52.9% in the model for men. These findings are in accordance with results observed in an equivalent sample of young cocaine users with the same instruments[34] and in contrast with some previous results where HRQL was not clearly related to some determinants of dependence, like amount and frequency of drug use[7]. Measuring severity of dependence directly with a validated instrument probably helped us to detect this relationship. Also the sample included a considerable heterogeneity of drug careers which can facilitate finding a significant result. In fact, 7% of the subjects had an SDS-H score of two or less, and for 50% it was higher than 8, also for SDS-C the corresponding figures were 35.6% and 24.4%.

Women showed worse HRQL, which is in accordance with studies in many different populations independently of the instrument used. In previous opiate-user groups gender differences in generic HRQL didn't achieve statistical significance[8,18] or only for some aspects of the SF-36[7]. Probably the sample size of the present study has helped to underline this difference. Furthermore, the large number of women included allowed a stratified analysis to be performed and construction of a multivariate model exclusively for them in which the set of variables found to be statistically significant differs from that of men. Besides SDS-H and SDS-C, only two other drug-related variables were retained in the female's model, daily alcohol intake and length of cocaine use. When doing the analysis with the total sample an interaction between gender and HIV infection was found, indicating that positive HIV serology only had an impact on HRQL of men. Some studies have found a slower progression to AIDS among HIV positive women, and Jarrin et al say that "in settings with small gaps in gender inequality and universal access to care, HIV-infected women fare better than their male counterparts in the era of HAART"[35].

Contrary to previous studies[14,34] poly-drug use was not confirmed as an independent factor for HRQL, not even when considering as a continuous variable the number of illegal substances used with a frequency of weekly or higher. Even though our variable was a proxy of DSM-IV poly-drug use, thus not directly comparable with other studies, it is worth signalling that it was not found to be related in a model in which the severity of cocaine dependence was an important independent HRQL predictor, thus somewhat accounting for another substance used and where, for the total sample and for women, daily alcohol intake was an independent factor positively associated with impaired HRQL. For males, recent overdoses, another factor related to poly-drug use, was also included in the model[36].

Poor health has been suggested, among other factors, as predisposing to heroin overdose[25]. In the present study subjects, especially males, who suffered an opiate overdose in the previous 12 months had an impaired HRQL. But, as this is a cross-sectional study it is not possible to know the direction of this association. Some authors consider specific systemic diseases like HIV, liver and lung disease as predisposing factors for overdose[26]. Those systemic diseases would by themselves affect HRQL, thus it would be difficult to unravel the precise causal path in the association between opiate overdose and HRQL. However, in the present study HIV and overdose were independently associated with HRQL. As some studies have also shown that, after an overdose, drug users have subsequent episodes of impaired health[37] the opposite sense of the association between poor HRQL and overdose has to be considered and its directionality elucidated in further studies. Previous findings reported higher frequency of overdose episodes among subjects with longer heroin use and higher severity of dependence[23]. The present study provides evidence that both overdose and severity of drug use are associated with poor perceived health as independent factors.

The study population was not gathered from treatment facilities, and although a large proportion of subjects had already contacted treatment services, their global NHP score was lower (better) than subjects starting treatment[8]. Nevertheless, within the study there was a gradient, subjects that had received drug treatment declared a worse HRQL than those who had not received it. Interestingly, after adjusting for all other relevant variables, subjects who in the last 12 months had received methadone treatment for their drug use, presented better HRQL. This is a remarkable finding as although more impaired subjects would be more prone to seek treatment[38], other variables explained the impaired HRQL to a point that having been in methadone treatment showed up as beneficial. This fact is consistent with the already ample evidence of methadone treatment effectiveness [39-41]. Other studies have proved the worth of treatment and a statistically significant improvement in HRQL has been demonstrated already after only one month in methadone maintenance[10].

We were not able to directly assess the influence of psychiatric comorbidity in HRQL, as it was not included among the variables studied at baseline. However, the fact of having received psychiatric treatment, which according to the study of a subsample of these subjects[42], was associated with psychiatric comorbidity, was one of the variables independently associated to the global score of NHP for males. This finding appears to lend further support for the relationship found in previous studies analysing psychiatric comorbidity and HRQL[19,20].

One socio-demographic factor related to HRQL, both in previous studies and in this group of young heroin users, was employment status, for which both males and females who worked exhibited better HRQL. However, in a cross-sectional study it is hard to say whether employment status is a consequence or a cause of impaired health. The other socio-demographic factor detected, educational level, was only significant for males and the overall sample, better-educated subjects presenting better HRQL. This is a factor that reflects inequalities in health and shows up once more in this population of young heroin users. Low educational level, one of the indicators used to assess inequalities in health, has been associated with increased mortality in different studies including intravenous drug user groups[43,44]. In the model for women alone it was not significantly related to HRQL probably because the distribution of this variable was more homogeneous than in men (i.e.: a lower proportion of women with primary studies not completed) and maybe to the smaller sample size.

## Conclusions

These heroin users were at a considerable risk of impaired health even at their young ages. HRQL was very much influenced by the severity of dependence, and improved with methadone treatment, thus specific interventions such as increasing effective drug treatment accessibility could improve HRQL of young heroin users.

## Competing interests

The authors declare that they have no competing interests.

## Authors' contributions

ADS participated in the design of the study, performed the statistical analysis and drafted the manuscript. MTB conceived of the study, participated in its design and coordination and helped to perform the statistical analysis. GB and MJB conceived of the study and helped to draft the manuscript. FGS participated in the design of the study and helped to draft the manuscript. LF conceived of the study, participated in its design and coordination and helped to draft the manuscript. All authors read and approved the final manuscript.
